# Reasons for Renal Donation among Living Unrelated Renal Donors in Khuzestan Province, Southwestern Iran

**Published:** 2013-02-01

**Authors:** S. S. Beladi Mousavi, M. J. Alemzadeh Ansari, A. Parsi, E. Kiani

**Affiliations:** 1 *Department of Internal Medicine, Faculty of Medicine, Jundishapur University of Medical Sciences, Ahvaz, Iran.*; 2 *Faculty of Medicine, Tehran University of Medical Sciences, Tehran, Iran*

## Abstract

Background: The shortage of living-related and cadaveric donors lead to living-unrelated kidney transplant in Iran.

Objective: We conducted this study to determine the motivations for unrelated-living kidney donation in Khuzestan province, southwestern Iran.

Methods: After obtaining an informed written consent, unrelated-living kidney donors were interviewed by the authors by means of a standardized questionnaire to assess their socioeconomic status and motivations for donation.

Results: 210 living kidney donors consisting of 167 men (79.5%) and 43 women (20.5%) with a mean±SD age of 28.4±5.6 years were studied. 117 (55.7%) donors were married. 6 (2.9%) of donors were university graduates; 39 (18.6%) high school graduates; 141 (67.1%) less than high school graduates; and 20 (9.5%) were illiterate. The motives for donation was mentioned mostly financial by 127 (60.5%) donors, mostly based on religious beliefs and altruism by 39 (18.6%), and a combination of financial, religious beliefs and altruism by 35 (16.7%) donors.

Conclusion: Financial problems are the main motivation for living-unrelated kidney donation in Khuzestan province, southwestern Iran.

## INTRODUCTION

It has been shown that kidney transplantation is the best replacement therapy for all patients with end-stage renal disease (ESRD), particularly for those with co-morbid diseases [1, 2]. In Iran, the prevalence and incidence of ESRD have increased respectively, from 238 and 49.9 per million people in 2000 to 357 and 63.8 per million people in 2006 [3, 4]. The first renal transplantation was performed in Iran from living donor in 1967. From 1967 to 2006, a total of 21,251 renal transplantations were performed, 78% of which were from living unrelated donors, 17% from living related, and 5% from deceased donors [5]. Kidney transplantation in Iran was started from living related donors mostly based on altruistic motives. However, the shortage of organs and absence of deceased donors have led to transplants from living unrelated donors [6] so that the main source of kidney donations in Iran is currently from living unrelated donors. The living unrelated donor renal transplantation has been carried out by Iranian model transplantation protocol since 1988 [5]. In earlier studies, various motives for kidney donation have been identified; the main motives mentioned were altruism, moral duty, religious beliefs, *etc *[7-9]. However, scarce information is available from Khuzestan, southwestern Iran about the motives of living unrelated kidney donors. We therefore conducted this study to examine this isuue.

## MATERIALS AND METHODS

This study was approved by the Ethics Committee of the Nephrology Research Center, affiliated to Ahvaz Joundishapur University of Medical Sciences. We designed a provisional questionnaire to assess the motives of the living unrelated kidney donors in our region. The questionnaire was tested in a pilot study. According to the results, the questionnaire was revised. The questionnaire along with an instruction were sent to Ahvaz Transplantation Center in 2008 to be distributed among all transplant donors before discharging from the hospital. The head nurse of the Transplant Center was in charge of administration of the questionnaire. The transplant nurse could help those donors who had problem with filling the questionnaire. Informed consent was obtained from all donors; they were assured that their responses were kept confidential. This study was continued until the end of 2009.

In the questionnaire, the donors were asked about their age, sex, nationality, relation with the recipient, education level, marital status, history of smoking, consumption of alcohol or opium, and the main reason for organ donation. Clinical data of the donors were also collected. The obtained data were then analyzed by SPSS^®^ ver 15 for Windows^®^, using χ^2^ and Fisher’s exact test when appropriate. Continuous variables were presented as mean±SD; categorical variables were presented as frequencies.

## RESULTS

Overall, 210 living kidney donors consisting of 167 (79.5%) men and 43 (20.5%) women were studied. The mean±SD age of all donors was 28.4±5.6 (range: 19–48) years. None of the donors had any known psychological disorders (Table 1). Most of the donors belonged to a low- or mid-socioeconomic class. Of 210 participants, 20 (9.5%) were illiterate, and 141 (67.1%) had educations less than high school.

**Table 1:** Demographic characteristics of 145 living kidney donors

Characteristics	n (%)
Sex	
Male	167 (79.5)
Female	43 (20.5)
Place of living	
Urban	183 (87.1)
Rural	27 (12.9)
Currently married	
Yes	117 (55.7)
No	93 (44.3)
Education	
Illiterate	20 (9.5)
Less than high school	141 (67.1)
High school degree	39 (18.6)
University degree	6 (2.9)
No answer	4 (1.9)
Smoke status	
Yes	83 (39.5)
No	111 (52.9)
No answer	16 (7.6)
using Alcohol	
Yes	7 (3.3)
No	179 (85.2)
No answer	24 (11.4)
using Opium	
Yes	15 (7.1)
No	172 (81.9)
No answer	23 (11.0)
Relation between donor and Recipient	
Related	21 (10.0%)
Un-related	189 (90.0%)

The motives for donation was mentioned mostly financial by 127 (60.5%) donors, mostly based on religious beliefs and altruism by 39 (18.6%), and a combination of financial, religious beliefs and altruism by 35 (16.7%) donors (Table 2).

**Table 2:** The main motivations of living related and unrelated kidney donors

Motivation	Related (n=21)	Unrelated (n=189)	Total (n=210)	p value
Financial	7 (33%)	155 (82.0%)	116 (77.3%)	<0.001
Religious beliefs	0	27 (14.3%)	19 (12.7%)	NS
Wish to help	14 (67%)	3 (1.6%)	12 (8.0%)	<0.001

The financial motives were significantly (p<0.001) more common among unrelated comparing to related donors whose incentive was mainly help and based on altruism (Table 2).

## DISCUSSION

Since the 1980s, many countries have passed legislation prohibiting monetary compensation for organ donation. Unfortunately, in practice altruistic motives for organ donation are far cry from adequate. During the past two decades, several approaches have been adopted to increase altruistic organ donations, but the gap between supply and demand has worsened in the due course. In 1988, a compensated and regulated living unrelated donor renal transplant program was adopted in Iran. Currently, Iran has no renal transplant waiting lists and as much as 50% of patients with ESRD in Iran are living with a functional graft [10].

The results of this study showing that most of the donors were unrelated are in keeping with those reported earlier from Iran [11-13]. Most of the studied donors had low- or mid-socioeconomic status; almost 10% were illiterate, and 67% had educations less than high school. Many studies have benn conducted on the socioeconomic status of donors and recipients. It seems that most unrelated donors do so for financial problems. Nevertheless, this is not limited to Iran, and financial problem was found to be the main motives for organ donation in many countries [11-15].

Zargooshi conducted a study on 300 kidney donors in Kermanshah, northwestern Iran and showed that the motives for donation was mainly financial in 43% of donors, and mainly financial with some altruism in 40% of donors [12]. Malakoutian, *et al*, conducted a study on 478 kidney donors from 30 transplant centers in Iran and showed that the financial issues were the most frequent motives for donation [13]. Heidary, *et al*, in a study on 721 kidney donors from 25 kidney transplantation centers in Iran, found that the motivations for donation were mostly financial in 37% of donors, and financial and altruism in 61% of donors [11]. Naqvi, *et al*, reported similar patterns in Pakistan; they found that of 239 studied kidney donors from Pakistan the most common reason for donation were financial problems in 72% of donors [17]. A cross-sectional study conducted in India revealed that almost all donors (96%) sold their kidneys to pay off debts. The most common sources of these debts were food and household expenses, rent, marriage expenses, and medical expenses [18].

Some studies from western communities revealed other motives for organ donation. Lennerling, *et al*, reported that of 154 donors (79 from Sweden and 75 from Norway), the strongest motives were based on altruistic grounds [19].

It was shown that ironically not only any economical improvements had been occurred in the life of those who donated organ for want of money but they also developed degrees of deteriorated general health. Naqvi, *et al*, showed that 88% of donors had no economic improvement in their lives and that 98% of them reported deterioration in general health status [20]. Goyal, *et al*, reported that the average income of donors family declined by one-third after nephrectomy; 86% of whom reported deterioration in their health status [21]. Zargooshi showed that vending caused negative impacts on the employment in 65% of donors—71% had severe *de novo* post-operative depression and 60% developed anxiety [22].

Although, the Iranian government pays for hospital admission and transplantation, and also provides essential immunosuppressive drugs freely, it does not support living kidney donors with a longterm medical insurance that can lead to the presence of dissatisfaction of donors. Therefore, we believe that it is necessary to establish a social network for support of kidney transplant donors in Iran.
